# Development and Validation of a Predictive Score for Preoperative Diagnosis of Early Stage Epithelial Ovarian Cancer

**DOI:** 10.31557/APJCP.2019.20.4.1207

**Published:** 2019

**Authors:** Watcharin Chirdchim, Preecha Wanichsetakul, Phichayut Phinyo, Jayanton Patumanond, Komsun Suwannarurk, Jatupol Srisomboon

**Affiliations:** 1 *Department of Obstetrics and Gynecology, Phrapokklao Hospital, Chanthaburi,*; 2 *Department of Obstetrics and Gynecology, Faculty of Medicine, Thammasat University, Pathum Thani, *; 3 *Research Division, Maesai District Hospital, Maesai, Chiang Rai *; 4 *Center for Clinical Epidemiology and Clinical Statistics,*; 5 *Department of Obstetrics and Gynecology Faculty of Medicine, Faculty of Medicine, Ching Mai University, Chiang Mai,Thailand. *

**Keywords:** Adnexal mass, ovarian cancer, prediction score, risk scoring

## Abstract

**Objective:**

To develop and validate a simplified multi-parameter risk-based scoring system for preoperative diagnosis of early stage epithelial ovarian cancer.

**Methods:**

All women presented with adnexal mass and were scheduled for operation at Phrapokklao hospital during September 2013 – December 2017 were included and categorized according to their histopathologic reports into early stage ovarian cancer groups and benign ovarian tumor groups. Multivariable logistic regression was used to explore for potential predictors. The selected logistic coefficients were transformed into risk-based scoring system. Internal validation was done with bootstrapping procedure.

**Results:**

A total of 270 participants were included in analysis and predictive model development, 54 in early stage ovarian cancer group and 216 in benign ovarian tumor group. Menopausal status, two abnormal ultrasound findings (presence of solid component or ascites), tumor size and serum CA-125 level were used for derivation of the scoring system. The score-based model showed area under ROC of 0.88 (95%CI 0.82-0.93). The developed scoring system ranged from 0 to 51 was classified into 3 subcategories for clinical practicability. The positive predictive values for the presence of early stage ovarian cancer were 2.07 (95%CI 0.43-6.05) for low risk patient, 29.13(95%CI 19.65-41.58) for moderate risk patient, and 95.45(95%CI 77.16-99.88) for high risk patient.

**Conclusion:**

This simplified risk-based scoring system for preoperative diagnosis of early stage ovarian cancer could aid general physicians or general gynecologists in evaluation of patients presenting with ovarian tumors and help gynecologic oncologists in management planning and prioritization of patients for operation.

## Introduction

Ovarian cancer is the second most common and the most lethal gynecologic cancer in Thailand (Wanapirak et al., 2006; Arun-Muthuvel et al., 2014). The incidence of ovarian cancer was estimated at 5.2 per 100,000 women per year (Khukaprema et al., 2009). During early stage of the disease the patient usually experience only mild to no symptoms, therefore most of ovarian cancer are detected within their advanced stage. To date, there was no effective screening tools for detection of early stage ovarian cancer, unlike papanicolaou smear in cervical cancer detection (Landis et al., 1999; Tongsong et al., 2009). 

In 2016, The American college of Obstetricians and Gynecologists (ACOG) recommended the use of the Risk of Malignancy Index (RMI) as a patient referral tool to gynecologic oncologist, given the RMI was higher than the cut-off value. Although previous studies showed high sensitivity and specificity of RMI in differentiating benign and malignant ovarian tumors, several ovarian cancer patients showed lower RMI whereas some with benign tumor showed higher RMI above the referral threshold. This misclassification causes risk to both groups of the patient. As advanced stage patients tended to have score that surpassed threshold, the diagnosis was therefore usually straightforward. Conversely, most prediction tools had troubles with discriminating early stage ovarian cancer from either sides, benign tumor or advanced stage cancer. 

Clear discrimination between early and advanced stage ovarian cancer is essential for proper management of the patient. Patient with early stage would be planned for complete surgical staging whereas the advanced stage patient would be prepared for cytoreductive surgery (Diasaia, 2012). 

Many preoperative prediction tools for ovarian cancer were consistently developed in many institutions e.g. The Risk of Malignancy Index I-IV (Jacobs et al., 1990; Tingulstad et al., 1996; Tingulstad et al., 1999; Yamamoto et al., 2009), The Risk of Malignancy Algorithms, an ultrasound prediction model developed by the International Ovarian Tumor Analysis (IOTA) study (Moore et al., 2009), Rajavithi-Ovarian cancer predictive score (Yanaranop et al., 2016) and algorithm with HE4, menopausal status and ultrasound findings (Wilailak et al., 2015). However, none of them can effectively distinguish between early and advanced stage ovarian cancer. This probably resulted from the inclusion of advanced stage cancer patient during the development of prediction tools. We hypothesized that limiting the domain of our study to patients who presented with adnexal mass and excluding all patients with advanced stage of disease or metastases could create the clinical decision rules that is able to distinguish between early stage ovarian cancer and benign ovarian tumors. 

The purpose of this study is to develop and validate a simplified multi-parameter risk-based scoring system for preoperative diagnosis of early stage Epithelial ovarian cancer, so that it can be used as a more precise referral tool for general gynecologists and minimized the risk of misclassification to the patient. 

## Materials and Methods

*Study design and setting*

The diagnostic prediction research with retrospective cohort design was conducted at Phrapokklao hospital, a university-affiliated referral hospital located in the eastern seaboard of Thailand with approximately one to two hundred operation for adnexal mass and ovarian tumor were performed annually. 

*Selection of participants*

**Figure 1:**
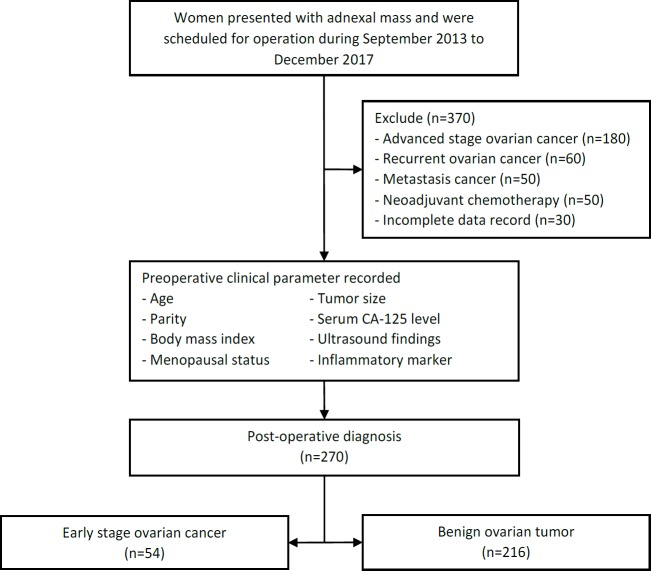
Study Flow

All women presented with adnexal mass and were scheduled for operation during September 2013 – December 2017 were included, regardless of types of operation performed. The eligible criteria were patients finally diagnosed with benign ovarian tumors and early stage Epithelial ovarian cancer according to postoperative tissue pathology report. The histopathologic reports were retrieved and reviewed for each included participant. The participants were then categorized into 2 groups according to their diagnoses, early stage ovarian cancer as an index group and benign ovarian tumors as a control group. Early stage ovarian cancer wasdefined as FIGO stage I, II, and III (microscopic) (citation FIGO staging). Due to limitation of frozen section report in our hospital, we included borderline ovarian tumor in the cancer group. The following were included as benign ovarian tumors: endometriotic cyst, mucinous cystadenoma, dermoid cyst, serous cystadenoma, tubo-ovarian abscess, fibroma, pseudo-cyst, and corpus luteal cyst. Diagnosis of advanced stage ovarian cancer, recurrent ovarian cancer, metastatic cancer, history of receiving neoadjuvant chemotherapy, and incomplete data record were excluded (Figure 1). 

*Model development and validation*

All clinical parameters necessary for diagnostic process in our routine practice were extracted from medical records including age, menopausal status, parity, body mass index, ultrasound findings by using ultrasound score, tumor size in centimeter(all ultrasound was done by attending staffs), inflammatory markers, and serum CA-125 level in U/ml. The ultrasound score was calculated by allocating 1 point for each abnormal ultrasonography features found e.g. Multilocularity, solid content, bilateral involvement of lesions, presence of ascites, and Intraabdominal metastases. The score was added up and transformed into 3 score categories (0 for no abnormality found, 1 for 1 abnormal feature found, and 3 for more than 2 abnormalities found. Potential predictors were selected based on prior knowledge from literature review and previous predictive models e.g. risk of malignancy index. Exploratory analysis of significant predictors was analyzed by using univariable logistic regression. Predictive significance of each predictor was justified by diagnostic odds ratio and its p value. Area under receiver operating characteristics (ROC) was also quantified for each of univariable logistic model. Predictive variables with diagnostic odds ratio more than 1.00, significant p-value of less than 0.05, and higher area under receiver operating characteristics than others were chosen for model development. Continuous potential predictors e.g. serum CA-125 level was transformed into ordinal variables in concordance to previous model’s categorization. 

Multivariable logistic regression was chosen for model derivation of study with binary outcome. Based on the univariate analysis, noncontributing factors were removed from the model. The total of five predictors were left in the reduced model which were menopausal status, tumor size, presence of solid components or ascites, and serum CA-125 level. The diagnostic accuracy of the reduced multivariable model was evaluated in term of calibration and discrimination. Measure of calibration was done with Hosmer-Lemeshow goodness of fit statistics. Calibration plot comparing agreement between the disease probabilities estimated via the model versus the observed disease data was also presented. Test of discriminative power was tested, visualized via distributional plot and reported with area under receiver operating characteristic curve. Internal validation was executed using bootstrapping procedure (1,000 replicates).

*Simplified risk score transformation*

Each item was then assigned with specific score derived from logistic regression coefficients of the multivariable model. The regression coefficient of each item was divided by the lowest coefficient, then rounded up to the nearest integer. The total score was then categorized into 3 risk groups (low, moderate, and high risk) for applicability in clinical practice. Due to population-analogue approach, positive predictive value (PPV) was calculated to present predictive performance separately for each risk category. The measurement of calibration and discrimination was also performed via score-based multivariable logistic model.

*Sample size calculation*

As there is no general census on the method of sample size calculation for derivation and validation studies for risk prediction models, we estimated the sample size by using two-sample comparison of means and proportions based on the previous study (Jacob,). Calculations were done for all of the potential predictors which were age, menopausal status, serum CA-125, tumor size, and ultrasound findings. The highest number of samples needed to achieve 90 percent statistical power and two-sided alpha error of 0.05 was calculated from menopausal status, requiring 111 for benign group and 34 for malignant group. In our study, all retrievable data were used to maximize the power and generalizability of the model derived. All data were analyzed by STATA version 14.1. (StataCorp, College Station, TX, USA). 

*Ethical consideration*

This study was approved by “The Research in Human Ethical Committee of Chanthaburi Province, document ID number CTIREC 006/61. Informed consents were not required in this retrospective data collection.

## Results

*Participants*

Six hundred and forty women were eligible for the study. Three hundred and seventy patients with advanced stage ovarian cancer, recurrent ovarian cancer, metastasis cancer, history of receiving neoadjuvant chemotherapy, and incomplete data recorded were excluded. A total of 270 participants were included in analysis and predictive model development, 54 in early stage epithelialovarian cancer group and 216 in benign ovarian tumor group (Figure1).

The participant in early stage ovarian cancer group had significantly older populations (48.7±15.4 vs. 43.5±12.1 years, p 0.009), higher proportion of postmenopause (51.9% vs. 19.0%, p < 0.001), larger tumor size (16.4±6.7 vs. 10.1±5.1, p < 0.001), higher serum CA 125 level (509.5±969.6 vs. 79.1 ± 158.4 u/mL, p 0.001), and higher proportion of abnormal ultrasound findings which comprised of the presence of solid component (61.1% vs. 26.4%, p < 0.001) and ascites (20.4% vs. 0.9%, p < 0.001). Risk of malignancy index calculated from the patient profile differed substantially among groups (509.5±2465.8 vs. 239±1607.0, p-value 0.005). Body mass index, parity status, and the presence of multilocularity from ultrasound finding showed no significant difference between groups. Among all clinical predictors, tumor size was the one with the highest predictive ability measured by the area under ROC (Table 1), followed by serum CA-125 level. 

*Model development*

After univariable logistic analysis, the menopausal status, two abnormal ultrasound findings (presence of solid component or ascites), tumor size and serum CA-125 level were combined in multivariable reduced logistic model for derivation of the scoring system. The area under ROC for the final model was 0.88 (95%CI 0.83-0.94) and p-value via Hosmer-Lemeshow goodness-of-fit test was 0.052. 

*Score transformation*

**Figure 2:**
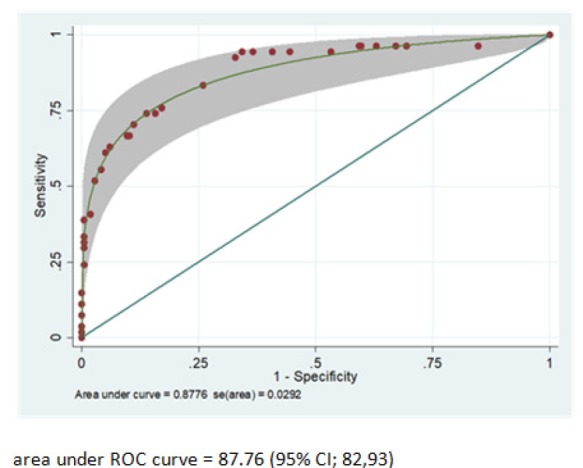
Performance of the Clinical Risk Score, Area under the Receiver Operating Characteristics (ROC) Curve and 95% Confidence Band

**Figure 3:**
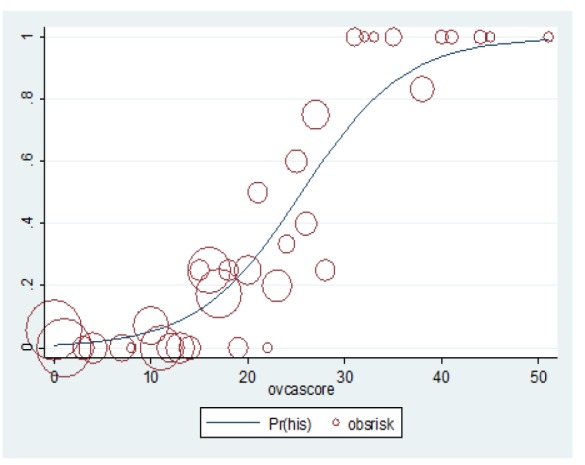
Observed Risk (Circle) vs Score Predicted Risk (Solid Line) of Ovarian Cancer. size of circle represent frequency of ovarian tumors in each score

Each potential predictor in the multivariable model was assigned with specific score derived from logistic regression coefficient (Table 2). The scoring scheme with a total score ranging from 0 to 51 was then further categorized into 3 risk subcategories for clinical applicability. This categorization was done based on the calibration plot between probability of having early stage ovarian cancer and score distribution, low risk group with score ranging from 0 to 14, moderate risk group with score ranging from 15 to 29 and high risk group with score ranging from 30 to 51. For discriminative ability, the area under parametric ROC for the score-based logistic regression model was 0.88 (95%CI 0.82-0.93) (Figure 2). Measure of calibration was illustrated with calibration plot and p-value goodness of fit of 0.056. From the plot, the predicted probability of early stage ovarian cancer increased as the score increased with high level of agreement between actual and predicted risks (Figure 3). The mean total score was significantly different between groups (27.0±1.5 vs. 10.3±0.6, p-value < 0.001). The positive predictive values were 2.07 (95%CI 0.43-6.05) for low risk, 29.13(95%CI 19.65-41.58) for moderate risk, and 95.45(95%CI 77.16-99.88) for high risk (Table 3). 

## Discussion

Nowadays, most of the preoperative prediction tools for ovarian cancer could not effectively distinguish patients with malignant tumors from patients with benign tumors. The risk of malignancy index (RMI) had been recommended and endorsed by many guidelines and institutions as the standard tool for clinical evaluation for patient with ovarian mass. RMI I-III relied on patient’s menopausal status, serum ca-125 level and ultrasound score (presence of solid component or ascites) for preoperative prediction of ovarian cancer (Jacobs et al., 1990; Tingulstad et al., 1996; Tingulstad et al., 1999). Meanwhile, in the latest revised version, RMI IV, tumor size was added to the model (Yamamoto et al., 2009). 

**Table 1:** Clinical Characteristics of Early Stage Ovarian Cancer vs benign Ovarian Tumor, Univariable Logistic Regression Odds Ratio, Evidence of Difference (p-value), Area Under Receiver Operating Curve with 95% Confidence Interval

Clinical Characteristics	Early stage ovarian cancer (n=54)		Benign ovarian tumor (n=216)		Odds ratio	P value	auROC
	mean	±SD	mean	±SD			
Age (years)	48.7	±15.4	43.5	±12.1	1.03 (1.01-1.06)	0.009	0.64 (0.54-0.73)
BMI (kg/m^2^)	23.8	±5.7	24.1	±4.7	0.99 (0.93-1.05)	0.678	0.46 (0.36-0.55)
Nulliparity (n, %)	21	(38.9)	69	(31.9)	1.36 (0.73-2.51)	0.334	0.53 (0.46-0.61)
Postmenopause (n, %)	28	(51.9)	41	(19)	4.60 (2.44-8.66)	<0.001	0.66 (0.59-0.74)
Tumor size (cm)	16.4	±6.7	10.1	±5.1	1.19 (1.11-1.26)	<0.001	0.80 (0.74-0.86)
Multilocularity (n, %)					1.16 (0.61-2.23)	0.649	0.52 (0.45-0.59)
Absence	16	(29.6)	71	(32.9)	-	-	-
Presence	38	(70.4)	145	(67.1)	-	-	-
Lesion involved (n, %)					0.41 (0.15-1.09)	0.075	0.45 (0.40-0.49)
Unilateral	49	(90.7)	173	(80.1)	-	-	-
Bilateral	5	(9.3)	43	(19.9)	-	-	-
Solid component (n, %)					4.38 (2.35-8.19)	<0.001	0.67 (0.60-0.75)
Absence	21	(38.9)	159	(73.6)	-	-	-
Presence	33	(61.1)	57	(26.4)	-	-	-
Ascites (n, %)					27.37 (5.86-127.91)	<0.001	0.60 (0.54-0.65)
Absence	43	(79.6)	214	(99.1)	-	-	-
Presence	11	(20.4)	2	(0.9)	-	-	-
Intraabdominal					8.27 (0.74-92.95)	0.087	0.52 (0.49-0.54)
metastasis (n, %)							
Absence	52	(96.3)	215	(99.5)	-	-	-
Presence	2	(3.7)	1	(0.5)	-	-	-
Ultrasound score (n, %)					1.60 (1.21-2.11)	0.001	0.63 (0.55-0.71)
0	5	(9.3)	34	(15.7)	-	-	-
1	21	(38.9)	124	(57.4)	-	-	-
3	28	(51.9)	58	(26.9)	-	-	-
Serum CA-125 (U/mL)	509.5	±969.6	79.1	±158.4	1.00 (1.00-1.00)	0.001	0.70 (0.62-0.79)
RMI score	2503.4	±5465.8	239	±1607.0	1.00 (1.00-1.00)	0.005	0.76 (0.68-0.84)

**Table 3:** Distribution of Early Stage Ovarian Cancer vs Benign Ovarian Tumor Across Different Level of Risk Categories (Low Risk, Moderate Risk, and High Risk), Positive Predictive Value (PPV), 95% Confidence Interval and p-value

Risk categories	Score	Early stage ovarian cancer (n=54)		Benign ovarian tumor (n=216)		PPV	95% Confidence interval	p-value
		n	%	n	%			
Low	<15	3	2.1	142	97.9	2.07	0.43-6.05	<0.001
Moderate	15-29	30	29.1	73	70.9	29.13	19.65-41.58	0.042
High	>29	21	95.5	1	4.5	95.45	77.16-99.88	<0.001
Mean±SE		27	±1.5	10.3	±0.6			<0.001

**Table 2:** Risk Score Derivation Using Multivariable Logistic Regression Coefficients

Potential predictors	Odds ratio	95% Confidence interval	p-value	Coefficients	Score
Menopausal status					
Premenopausal	1	reference	-	-	0
Postmenopausal	3.53	1.55-8.07	0.003	1.262611	7
Tumor size (cm)					
< 9	1	reference	-	-	0
9-12	6.38	1.50-27.12	0.012	1.853163	10
> 12	20.12	5.15-78.60	<0.001	3.001914	16
Solid component					
Absence	1	reference	-	-	0
Presence	1.89	0.83-4.32	0.13	0.6379465	3
Ascites					
Absence	1	reference	-	-	0
Presence	11.09	1.39-88.38	0.023	2.405997	13
Serum CA-125 level (U/ml)					
< 30	1	reference	-	-	0
30-200	1.21	0.49-2.96	0.681	0.1882698	1
> 200	9.11	2.86-29.06	<0.001	2.209166	12

In our study, we developed the simplified clinical risk score for preoperative prediction of early stage ovarian cancer based primarily on predictive parameters from RMI. During exploratory analysis we found that postmenopausal status, abnormal ultrasonographic features, presence of solid component or ascites, showed statistically significant diagnostic odds ratio for prediction of early stage ovarian cancer, and thus were included in our model. Many studies also reported high predictive ability of ovarian cancer from the presence of solid component within the mass (Moore et al., 2009; Yanaranop et al., 2016). 

Serum CA-125 is the most extensively investigated ovarian cancer associated tumor marker. CA-125 can be synthesized from any ovarian tumors, regardless of their malignant status (Hellerstrom et al., 2008). The elevation of serum CA-125 levels has been reported in 80% to 85% of patients with ovarian cancer at the time of diagnosis (Myers et al., 2006). The standard cut-off point of serum CA-125 for having higher probability of ovarian cancer was 35 U/mL for postmenopause women and 200 U/mL for women in their reproductive age (Rauh-Hain et al., 2015). However, in our diagnostic model, we categorized serum CA-125 into 3 groups (< 30 U/mL, 30-200 U/mL and > 200 U/mL) which was the most appropriate categorization that yielded the most diagnostic value thanks to the distribution of observations in our derivation cohort. 

The strength of this study was the inclusion of tumor size in the prediction model unlike RMI I-III or other scoring system. The size of tumor was categorized into 3 subcategories according to distribution of our data to increase as much diagnostic ability within the model. Our study showed higher proportion of patients with large tumor size (≥ 7 cm) than that reported in study by Yamamoto (98% vs. 85%), which might result from the inclusion of borderline ovarian tumor in early stage cancer group. Although borderline ovarian tumor patients received little survival beneﬁt from comprehensive surgical staging, many studies still recommend that borderline ovarian tumor patients should undergo initial surgical staging (Song, 2013). 

Although Wilailak et al., (2015) (AuROC 89.3%) and Yanaranop et al., (2016) (AuROC 92.8%) reported the use of HE-4 as a predictive parameter, we did not insert HE-4 within our model because it was not widely used in our clinical setting due to limited expenses, and usually required longer waiting time for the result. With categorization of continuous variables and exclusion of variables from previously reported models, five parameters were left within our scoring scheme. The predictive ability determined by area under ROC was still high and acceptable at 88 percent. The main limitation of our study was that all the data were retrospectively collected. Future prospective design could improve reliability and predictive ability of the model. Although internal validation was done in our study, the reproducibility of the risk scoring was still unknown until external validation study was executed on other setting or in different time dimension. 

Unlike other prediction tools, this new scoring system classified patients with ovarian tumor into 3 groups (low risk, moderate risk, and high risk) to practically guide physicians and general gynecologist in decision making in their routine practice. For group with low risk, patient could be managed entirely by gynecologists. Minimally invasive or laparoscopic surgery could be suggested as alternative to open surgery. Patient with moderate to high risk predicted by our model should be refereed to gynecologic oncologists for proper urgent surgical management plans. Intraoperative histopathologic consultation or frozen section should be done for both groups. The score also facilitates the prioritization of patients for operation according to their risk.

In conclusion, this simplified risk-based scoring system for preoperative diagnosis of early stage ovarian cancer could aid general physicians or general gynecologists in evaluation of patients presenting with ovarian tumors and help gynecologic oncologists in management planning and prioritization of patients for operation.

## Statement conflict of Interest

The authors declare no conflicts of interest.

## Funding Statement

None.
